# Mulcom: a multiple comparison statistical test for microarray data in Bioconductor

**DOI:** 10.1186/1471-2105-12-382

**Published:** 2011-09-28

**Authors:** Claudio Isella, Tommaso Renzulli, Davide Corà, Enzo Medico

**Affiliations:** 1 Department of Oncological Sciences, University of Torino, Str. Prov. 142 Km. 3.95, Candiolo, 10060 Italy; 2 Laboratory of Oncogenomics, Institute for Cancer Research and Treatment, Str. Prov. 142 Km 3.95, Candiolo, 10060 Italy; 3 Laboratory of Systems Biology, Institute for Cancer Research and Treatment, Str. Prov. 142 Km. 3.95, Candiolo, 10060 Italy; 4 Center for Molecular Systems Biology, University of Torino, Via Acc. Albertina, 13, Torino, 10023 Italy

## Abstract

**Background:**

Many microarray experiments search for genes with differential expression between a common "reference" group and multiple "test" groups. In such cases currently employed statistical approaches based on *t*-tests or close derivatives have limited efficacy, mainly because estimation of the standard error is done on only two groups at a time. Alternative approaches based on ANOVA correctly capture within-group variance from all the groups, but then do not confront single test groups with the reference. Ideally, a *t*-test better suited for this type of data would compare each test group with the reference, but use within-group variance calculated from all the groups.

**Results:**

We implemented an R-Bioconductor package named Mulcom, with a statistical test derived from the Dunnett's *t*-test, designed to compare multiple test groups individually against a common reference. Interestingly, the Dunnett's test uses for the denominator of each comparison a within-group standard error aggregated from all the experimental groups. In addition to the basic Dunnett's *t *value, the package includes an optional minimal fold-change threshold, *m*. Due to the automated, permutation-based estimation of False Discovery Rate (FDR), the package also permits fast optimization of the test, to obtain the maximum number of significant genes at a given FDR value. When applied to a time-course experiment profiled in parallel on two microarray platforms, and compared with two commonly used tests, Mulcom displayed better concordance of significant genes in the two array platforms (39% vs. 26% or 15%), and higher enrichment in functional annotation to categories related to the biology of the experiment (p value < 0.001 in 4 categories vs. 3).

**Conclusions:**

The Mulcom package provides a powerful tool for the identification of differentially expressed genes when several experimental conditions are compared against a common reference. The results of the practical example presented here show that lists of differentially expressed genes generated by Mulcom are particularly consistent across microarray platforms and enriched in genes belonging to functionally significant groups.

## Background

A frequent approach to analyse gene expression data involves the use of *t*-tests, or their derivatives, to identify lists of genes with differential expression between two experimental groups [1]. Indeed, several microarray expression datasets encompass multiple experimental points to be compared with a common reference point such as time-course designs or multiple different treatments versus a control condition. The analysis is then aimed at assessing for each gene in which experimental group the expression is significantly different from the control group.

A frequently chosen approach is to run a *t*-test for each comparison. However, when applied to this type of data, the *t*-test has two main problems: (i) it does not correct the result of each comparison for the total number of comparisons made and (ii) information about experimental variability (the standard error) is extracted only from the two groups actually compared. Consequently, in the instance of limited replicates, inaccurate estimation of standard error leads to high type I and type II errors in the analysis. For these two reasons, simple remedies like Bonferroni or other types of multiple testing correction of the threshold *t*-value may avoid excessive false positives only at the cost of a significant reduction of the power. Alternatively, limitations of the *t*-test in this context have been addressed by implementing Bayesian modeling of the error [2] or by implementing sample permutation-based estimation of False Discovery Rate (FDR), like in the SAM approach [3,4]. In particular, SAM compares the number of null hypothesis rejections in the dataset organized in the subgroups of interest against the median number of rejections on a series of randomly generated subgroups. These approaches however do not benefit, within each comparison, from information on within-group variability available in the additional experimental groups.

As an alternative, ANOVA-based methods accumulate within-group variability from all the groups. However, this strategy does not permit a pair-wise comparison of each test group with the reference group [5]. Therefore, if a gene is differentially expressed in only one group versus the reference, this difference is diluted in the between-group variance calculated from all the groups.

The ideal approach would therefore be to estimate within-group variance from all the groups and then to perform single pairwise comparisons. Towards this end, we designed the Mulcom test, a derivative of the Dunnett's *t*-test [6] adapted to microarray data analysis. The test, implemented as an R-Bioconductor package [7], includes an optional tuneable fold-change threshold (*m*) and Monte Carlo simulation performing sample permutations to assess FDR in each comparison. We also implemented a streamlined procedure for automated optimization of test parameters, to maximize the number of significant genes at a given FDR.

In the present work we provide a detailed description of the Mulcom algorithm and the results of comparative analyses between Mulcom and other widely used Bioconductor packages (SAM and Limma). Comparative analyses were run on a microarray dataset obtained on two different array platforms from the same set of samples.

## Implementation

The Mulcom test was implemented using the statistical programming language R [8] with some functions wrapped from C++ to improve the performance of the script. The package is included in the open-source Bioconductor project [7].

The Mulcom package is designed to analyse ExpressionSet objects from the "Biobase" package as well as standard numeric matrices from the R environment. The Mulcom algorithm is based on the Dunnett's t-test [6], which estimates the within-group variability across all the different groups to be compared with the common reference.

The Mulcom analysis takes place according to the following steps:

For each experimental group E, it compares the average signal *E *with that of the common reference group *C *to obtain the function.

(1) $$FC=\bar{E}-\bar{C}$$

It then applies all the experimental groups to calculate the within-group Mean Square Error (*MSE*):

(2) $$MSE_{wg}=\frac{\sum \overset{\text{2}}{\underset{i}{s}}}{a},$$

where *s*^2 ^= square error, for each group *i*, including the reference group and *a *= degrees of freedom

Finally, the Mulcom *t *is calculated:

(3) $$t=\frac{|FC|-m}{\sqrt{\frac{\text{2}*MSE_{wg}}{Nh}}}$$

Where:

*FC *= fold change (difference), as calculated in (1)

*m *= minimal difference threshold (optional)

Nh = harmonic mean of sample replicates for the two conditions tested

*t *= *t*-value of the test

*MSE_wg _*= mean square error within group, as calculated in (2)

To estimate the False Discovery Rate (*FDR*), steps (1) to (3) are repeated after random sample permutation for *n *times, to generate a distribution of the number of positive hits from *n *randomly assembled sample groups.

For each experiment-to-reference comparison, the median number of positives in permuted sample groups is calculated, and FDR is estimated as $FDR=\frac{\bar{MRP}}{EP}$

Where:

*MRP *= Median Random Positives, i.e. the median number of null hypothesis rejections by the Mulcom test in all random sample permutations.

*EP *= Experimental Positives.

**Figure 1:**
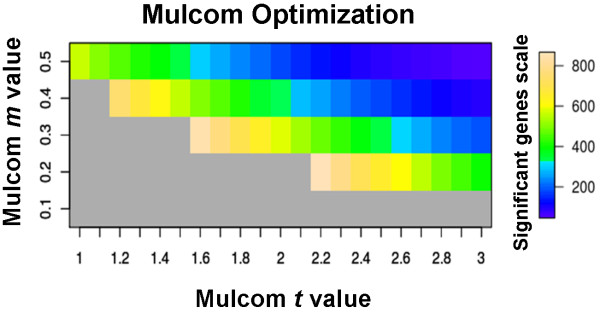
**Mulcom optimization plot**. Optimization plot generated by Mulcom to choose test parameters (*m *and *t*). The heatmap highlights the number of significant genes for each combination of *m *(*y*-axis) and t (*x*-axis) with a FDR below the threshold defined by the user (five percent in this case). Given the FDR threshold of choice, the box with the colour closest to the top of the scale indicates *t *and *m *values giving the maximum number of significant genes. Additional boxes to the right and top of the lightest one are shown to provide an estimate of the number of significant genes passing the test under more stringent *t *and *m *conditions. Whether such conditions further improve FDR, should be tested by fixing a lower FDR threshold and repeating the analysis.

If no *m *fold change threshold is applied, users can manually define *t*-value significance thresholds on the basis of Dunnett's test alpha tables, such as the one at http://davidmlane.com/hyperstat/table_Dunnett.html. The degrees of freedom are obtained by subtracting the number of groups (including the control) from the total number of subjects in all groups. If the alpha tables cannot be used the package implements a set of functions to choose the best combination of *t *and *m*, i.e. one giving the highest number of positives at a chosen FDR rate. Furthermore the package also assists the user in the identification of alternative combinations of *t *and *m*, which can be evaluated and chosen using the Mulcom Optimization Plot (Figure 1). The plot visualizes the number of significant genes for each combination of *t *and *m *within the respectively chosen ranges, limiting the display to those combinations having an FDR below a threshold of choice. Together with the optional *m *threshold value for fold-change, the FDR analysis based on Monte Carlo simulation is the main difference between the Mulcom test and the conventional Dunnett's t-test. Additional information on the use of Mulcom is provided with the package vignette http://bioconductor.org/help/bioc-views/2.8/bioc/html/Mulcom.html.

## Results and Discussion

A previous spreadsheet-based implementation of the Dunnett's *t*-test was successfully applied to gene expression studies comparing multiple independent points against a common reference [9,10]. To evaluate the performance of the Mulcom test implemented as a Bioconductor package, we generated and analyzed transcriptomics data on a set of 10 RNA samples profiled with two independent microarray platforms (Affymetrix hgu133a and Illumina RS-8 Human Beadchip). This enabled cross-platform concordance analysis of the results. The experiment explored gene expression changes induced in MDA-MB-435 human melanoma cells by 1, 6 or 24 hours of stimulation with Hepatocyte Growth Factor (HGF), known to trigger proliferation, motility and invasion [11]. The same cells were also transduced with Integrin-Beta4 (ITGB4) to stably up-regulate its expression. Therefore the dataset encompassed both a time-course experiment and one positive control condition, each repeated to generate biological duplicates. Data were normalized and filtered for significant detection as described in the Preprocessing section.

**Figure 2:**
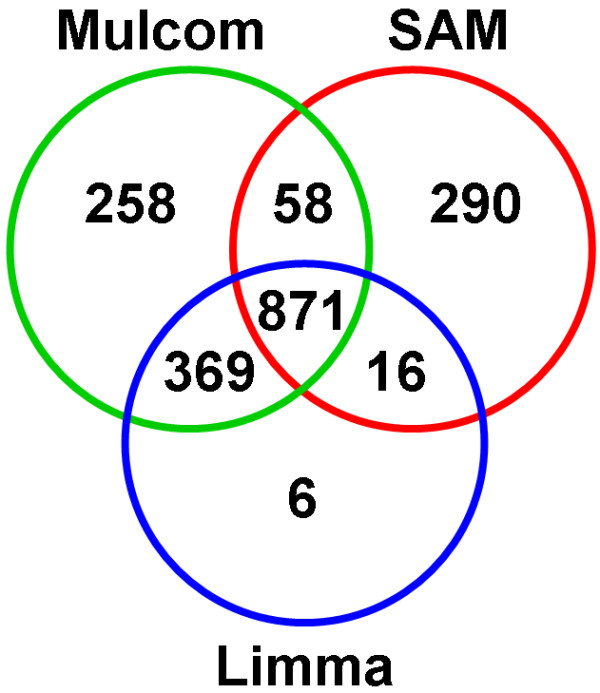
**Overlap between Mulcom and other tests**. Venn diagram showing intersections between lists of significant probe sets defined by Mulcom, SAM and Limma (Affy dataset). These show a limited partial but significant overlap.

Mulcom analysis on Affymetrix data identified a total of 1556 significant probe sets (1249 genes), out of 10137 detected probe sets (8105 unique genes), at a threshold FDR below 5%. The same data were analyzed with Bioconductor implementations of two other widely used tests, Limma and SAM [2-4], each tuned to yield a comparable number of significant probe sets. SAM identified 1235 probe sets (1006 genes) with FDR below 1%, and Limma identified 1262 probe sets (956 genes) with p-value below 0.05. The intersection between the three lists was of 871 probe sets, showing a notable but partial concordance (Figure 2). All Affymetrix probe sets were then mapped to the Illumina dataset by gene symbol, and the three tests were applied to this second dataset, maintaining the respective FDR or p-value thresholds, to check cross-platform consistency. Interestingly, Mulcom displayed the highest fraction of validated genes (Table 1) and a similar number of significant genes in the two platforms.

**Table 1:** Validation across microarray platforms of Mulcom, Limma and SAM tests

		Mulcom	Limma	SAM
**HGF 1 h**	Significant genes in Affy	867	672	723
	Validated in Illumina	150	0	48

**HGF 6 h**	Significant genes in Affy	681	518	561
	Validated in Illumina	317	237	100

**HGF 24 h**	Significant genes in Affy	4	0	82
	Validated in Illumina	1	0	1

**ITGB4 +/-**	Significant genes in Affy	26	6	75
	Validated in Illumina	1	1	0

**Total**	Significant genes in Affy	1249	956	1006
	Validated in Illumina	487	246	151
	True positive rate	39%	26%	15%

Number of genes identified by Mulcom, Limma and SAM tests in the Affymetrix dataset, and the number of validated genes in the Illumina dataset for all the pair-wise comparisons.

**Figure 3:**
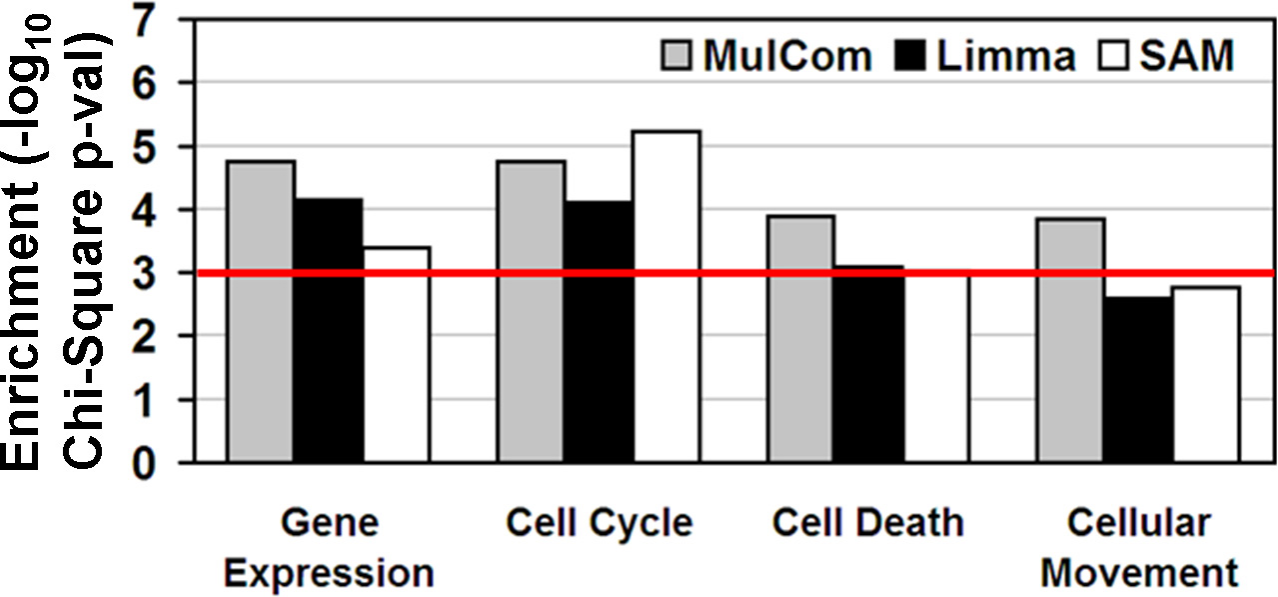
**Functional significance of Mulcom results**. Enrichment in annotation to specific cellular functions for gene lists generated by Mulcom, SAM and Limma (p-value below 0.001) and analysed using the Ingenuity pathway.

To assess the functional significance of the genes identified by the three tests, we analysed them using Ingenuity Pathways Analysis (Ingenuity^® ^Systems, http://www.ingenuity.com. For each of the three tests, the list of significant genes defined in Affymetrix data was tested for enrichment in specific functional annotation keywords. Four keywords displayed an enrichment p-value below 0.001 in at least one of the three lists: "gene expression", "cell cycle", "cell death" and "cellular movement". Interestingly, these categories altogether recapitulate the biological effects of HGF on epithelial and other adherent cells. As shown in Figure 3, the Mulcom list was significant in all four categories, and the most significant in three of them. We then repeated this analysis on lists generated by the three tests on the Illumina dataset. Overall, enrichments were lower, and therefore the significance threshold was lowered to p < 0.005, yielding nine keywords enriched for Mulcom, seven for Limma and three for SAM (data not shown).

To extend the significance of the results we performed the same comparison between Mulcom, Limma and SAM on an unrelated time course experiment (GSE19044) [12]. In this experiment, germ line cell-derived pluripotent stem cells (GPSC) were induced to differentiate into hepatocytes and subsequently profiled at different stages. In order to identify differentially regulated genes between the reference time 0, and the different temporal stages, (namely day 2, day 7 day 21 and day 27) we applied the same settings for the statistical analysis as previously described (FDR < 0.01 or corrected p-value < 0.01) on the data series GPSC-A and GPSC-B (two independent cell lines). Cross validation of the results highlighted that Mulcom test was the most efficient in identifying a high number of differentially regulated genes, which were systematically validated in the second experiment. The results are presented in Additional file 1, Figure S1.

## Conclusions

Overall, these results show that, in a multiple comparison setting, the Mulcom package is particularly good at generating reliable lists of biologically informative genes. In our opinion, the main reasons for the good performance of Mulcom under these conditions are as follows: (i) Within-group variability is estimated from all experimental groups even if only two of them are compared at a time. It is therefore more reliable when few replicates are available for each group; (ii) The optional fold-change threshold *m *avoids false positives due to aberrantly low within-group variability and (iii) Automated test optimization linked to permutation-based FDR analysis allows sensitivity to be maximised without compromising specificity. Indeed, such an approach could be prone to overfitting i.e. identification of apparently optimal settings, which are highly dependent on the dataset. Of particular relevance to this issue is the fact that in the above-described dual platform-dataset the Mulcom test, albeit having been separately optimized on each of the two microarray platforms, yielded the most concordant lists of significant genes. Mulcom can also easily be applied to other -omics studies, like miRNomics, proteomics and metabolomics, where multiple experimental points are compared against the same reference.

## Preprocessing

### Microarray Data generation and preliminary treatment

Gene expression profiling was performed on the same set of RNAs independently on Affymetrix hgu133a and Illumina Human 8-V1 arrays, according to the manufacturer's protocols. Affymetrix raw data were processed with the R-Bioconductor suite http://www.bioconductor.org. Technical quality analysis was performed with the "Affy" package [13]. Probe data was summarized and normalized with RMA [14] Probe sets without a positive presence call in at least two samples were excluded from further analyses. Illumina data were processed with the BeadStudio software 1.5.13 (Illumina) with Rank Invariant Normalization. Probes for which all samples showed a Detection Score lower than 0.99 were excluded from further analyses. Raw and normalized microarray data from both platforms are deposited in NCBI's Gene Expression Omnibus repository (accession ID: GSE26736).

### Ingenuity Pathway Analysis

All lists of gene symbols generated by the various tests were uploaded on IPA http://www.ingenuity.com and tested for enrichment in molecular and cellular functions. Enrichment (chi-square p-value) was estimated against the MDA-MB-435 background provided by the IPA software.

## Availability and requirements

Project name: Mulcom

Project home page: Operating system(s): Platform independent

Programming language: R

License: GNU GPL

Any restrictions to use by non-academics: none

Availability: http://bioconductor.org/help/bioc-views/2.8/bioc/html/Mulcom.html

## List of abbreviations

EP: Experimental Positives; FC: Fold Change; FDR: False Discovery Rate; GPSC: germ line cell-derived pluripotent stem cells; HGF: Hepatocyte Growth Factor; IPA: Ingenuity Pathway Analysis; ITGB4: Integrin-Beta4; MRP: Median Random Positives; MSE: Mean Square Error; SAM: Significance Analysis of Microarray

## Authors' contributions

EM and CI conceived the Mulcom Test, CI conceived the package and performed all the analyses. DC and TR developed part of the code. CI, TR, DC and EM wrote the manuscript. All authors read and approved the final manuscript.

## Supplementary Material

Additional file 1 **Figure S1: Comparison between Mulcom, Limma and SAM on a time-course stem cell differentiation dataset**. Two time-course series conducted in parallel have been analyzed, A and B. Blue, red and green columns indicate the number of significant genes at each time point detected by, respectively, Mulcom, Limma and Sam. Internal columns in light blue, orange and light green indicate the number of genes significant in series A that were also significant in series B as identified by, respectively, Mulcom, Limma and SAM. Click here for file
