# Supplementary figures and images for: Mulcom: a multiple comparison statistical test for microarray data in Bioconductor
Source: BMC Bioinformatics. 2011 Sep 28;12:382. doi: 10.1186/1471-2105-12-382 (PMC3230912; doi:10.1186/1471-2105-12-382)

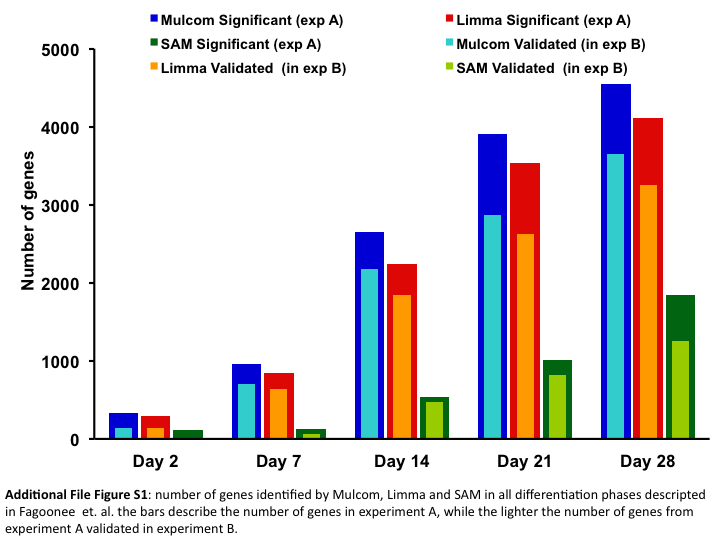

Supplement: Additional file 1 — Figure S1: Comparison between Mulcom, Limma and SAM on a time-course stem cell differentiation dataset. Two time-course series conducted in parallel have been analyzed, A and B. Blue, red and green columns indicate the number of significant genes at each time point detected by, respectively, Mulcom, Limma and Sam. Internal columns in light blue, orange and light green indicate the number of genes significant in series A that were also significant in series B as identified by, respectively, Mulcom, Limma and SAM. [file 1471-2105-12-382-S1.PNG]
